# *mazEF* Homologue Has a Minor Role in *Staphylococcus epidermidis* 1457 Virulence Potential

**DOI:** 10.3389/fcimb.2021.803134

**Published:** 2022-01-13

**Authors:** Vânia Gaio, Tânia Lima, Manuel Vilanova, Nuno Cerca, Angela França

**Affiliations:** ^1^ Laboratory of Research in Biofilms Rosário Oliveira, Centre of Biological Engineering, University of Minho, Braga, Portugal; ^2^ Instituto de Investigação e Inovação em Saúde, Universidade do Porto, Porto, Portugal; ^3^ Instituto de Biologia Molecular e Celular, Universidade do Porto, Porto, Portugal; ^4^ Instituto de Ciências Biomédicas de Abel Salazar, Universidade do Porto, Porto, Portugal

**Keywords:** *SERP1681*, *SERP1682*, antimicrobial tolerance, biofilms, human blood, human plasma, macrophages, dendritic cells

## Abstract

*Staphylococcus epidermidis* biofilm cells are characterized by increased antimicrobial tolerance and improved ability to evade host immune system defenses. These features are, in part, due to the presence of viable but non-culturable (VBNC) cells. A previous study identified genes potentially involved in VBNC cells formation in *S. epidermidis* biofilms, among which *SERP1682/1681* raised special interest due to their putative role as a toxin–antitoxin system of the *mazEF* family. Herein, we constructed an *S. epidermidis* mutant lacking the *mazEF* genes homologues and determined their role in (i) VBNC state induction during biofilm formation, (ii) antimicrobial susceptibility, (iii) survival in human blood and plasma, and (iv) activation of immune cells. Our results revealed that *mazEF* homologue did not affect the proportion of VBNC cells in *S. epidermidis* 1457, refuting the previous hypothesis that *mazEF* homologue could be linked with the emergence of VBNC cells in *S. epidermidis* biofilms. Additionally, *mazEF* homologue did not seem to influence key virulence factors on this strain, since its deletion did not significantly affect the mutant biofilm formation capacity, antimicrobial tolerance or the response by immune cells. Surprisingly, our data suggest that *mazEF* does not behave as a toxin–antitoxin system in *S. epidermidis* strain 1457, since no decrease in the viability and culturability of bacteria was found when only the *mazF* toxin homologue was being expressed.

## Introduction

*Staphylococcus epidermidis*, a commensal bacterium that is a common inhabitant of the skin and mucous membranes of humans and several mammals, is recognized by its ability to form thick and multi-layered biofilms, especially on the surface of indwelling medical devices (Fey and Olson, 2011). Although *S. epidermidis* generally presents a benign relationship with the host, this species has been considered an opportunistic pathogen particularly affecting preterm neonates and immunocompromised patients and is now considered one of the main causes of medical device-associated infections (Otto, 2009).

Previous *in vitro* studies have shown that *S. epidermidis* biofilms present an increased tolerance to antibiotics (Cerca et al., 2005; Anderson and O’Toole, 2008) and to the host immune response (Brescó et al., 2017; Nguyen et al., 2017), which may hinder the treatment of biofilm-associated infections. Moreover, *S. epidermidis* cells can enter a state of dormancy, either becoming persisters (Shapiro et al., 2011) or viable but non-culturable (VBNC) (Zandri et al., 2012). The presence of high amounts of VBNC cells can hamper the routine detection or interpretation of the actual status of the infection (Zandri et al., 2012). Furthermore, VBNC cells within *S. epidermidis* biofilms account for a greater ability to evade the host innate immune system (Cerca et al., 2011a) and decreased susceptibility to antibiotics (Cerca et al., 2014).

Following an *in vitro* model that induces VBNC cells in *S. epidermidis* biofilms (Cerca et al., 2011a), the transcriptome of biofilms with higher amounts of VBNC cells was characterized. Among the genes of interest identified, *SERP1682*, a *mazE* homologue, raised special interest since it was uniquely detected when the VBNC state was induced (Carvalhais et al., 2014). Importantly, the *mazEF* gene family has been described as a toxin–antitoxin (TA) system and is composed of genes that encode a stable toxin (*mazF*) and a labile antitoxin (*mazE*) (Van Melderen, 2010), which are involved in the regulation of essential cell processes (Karimi et al., 2015). These genes were first described as a TA system in *E. coli* (Metzger et al., 1988). Nevertheless, the function of this module as a TA system has been experimentally confirmed in other species such as *Streptococcus mutans* (Syed et al., 2011), *S. aureus* (Zhu et al., 2009), and *S. equorum* (Schuster et al., 2013). Furthermore, several homology studies indicate the presence of the putative *mazEF* TA complex in other species, such as *Pseudomonas aeruginosa* (Valadbeigi et al., 2017), *Mycobacterium tuberculosis* (Tiwari et al., 2015), and *S. epidermidis* (Behrooz et al., 2018). However, so far, the role of *mazEF* homologue in *S. epidermidis* virulence has not been determined. As such, herein, we constructed and characterized a *mazEF* homologue mutant strain.

## Material and Methods

### Strains and Growth Media

*S. epidermidis* 1457 (Mack et al., 1992) was used in this study. The wild type (WT) and constructs were grown overnight in Tryptic Soy Broth (TSB, Merck) at 37°C and with agitation at 120 rpm. In the case of strains harboring the plasmids pRB473 or pRMC2, TSB supplemented with 10 μg/ml chloramphenicol was used (TSB_CM10_). For further experiments, the overnight cultures were diluted in TSB or TSB_CM10_ to an optical density (OD) of 0.250 ± 0.05 at 640 nm, corresponding to approximately 2 × 10^8^ colony-forming units/ml (CFU/ml) (Freitas et al., 2014).

### Mutant Construction

Genetic manipulations were based on previous protocols (Brückner, 1997; Monk et al., 2012). Briefly, the flanking regions of the *mazEF* homologue operon were amplified and linked together by an overlap PCR. The resulting fragment was ligated into the plasmid pIMAY and transformed into *E. coli* DC10B, where the correct sequence and orientation of the flanking regions were confirmed by sequencing. The knockout plasmid was then transformed into the WT strain by electroporation (Monk et al., 2012; Cui et al., 2015). The allelic replacement protocol was performed as described in Monk et al. (2012) and the confirmation of the deletion of the *mazEF* homologue operon was done by PCR. Then, the *ΔmazEF* mutant was transformed with two distinct plasmids: the pRB473 (Bruckner et al., 1993) and the pRMC2 inducible plasmid (Corrigan and Foster, 2009). In brief, the homologous sequences of the genes *mazE*, *mazF* or the operon *mazEF* were amplified and cloned into pRB473 (sequences cloned included the target genes and their natural promoter region) or pRMC2 (sequences cloned included a strong ribosomal binding site and start and stop codons). While the plasmid pRB473 was first transformed into *E. coli* DC10B, the inducible plasmid pRMC2 was first transformed into *S. aureus* RN4220. Finally, the expression plasmids were inserted into *ΔmazEF* mutant strain by electroporation. The successful restoration of *mazE/mazF/mazEF* homologues expression was performed by qPCR, as described below. The primers used herein were designed using Primer3 software (Untergasser et al., 2012) and are listed in **Supplementary Table 1**.

### Induction of *mazEF* Expression

The optimal conditions to induce the pRMC2 plasmid were first assessed by evaluating the effect of different concentrations of anhydrotetracycline (ATC, 0.64 and 1.28 μg/ml) or tetracycline (TET, 0.08 and 0.16 μg/ml), both purchased from Sigma-Aldrich, in the culturability of *ΔmazEF* cells. Thereafter, the induction of the expression of the genes cloned into the inducible plasmid (*mazE, mazF* or *mazEF* homologues) was started by adjusting the overnight cultures of each construct to an OD_640nm_ = 0.5, followed by a 1:10 dilution in 10 ml of TSB_CM10_ and incubation for 3 h at 37°C and 120 rpm. Then, 0.64 μg/ml of ATC was added, and the tubes were incubated under the same temperature and agitation conditions for up to 24 h. A tube without ATC was used as a control. Aliquots were collected at the beginning of the induction (T = 0 h), after 30, 60, 90, 120, 180, and 240 min and at the end of the experiment (T = 24 h) to measure the OD and quantify the number of culturable cells.

### Planktonic Growth and Biofilm Formation

A suspension with approximately 2 × 10^8^ CFU/ml, prepared as described above, was used to start both planktonic cultures and biofilms. Planktonic cultures were started by inoculating 100 μl of this suspension into 10 ml of TSB/TSB_CM10_ and further incubated at 37°C and 120 rpm, for up to 24 h. Biofilms were formed as described before in either 24-well plates (for VBNC state assessment, confocal analysis and gene expression studies) (Cerca et al., 2011a; Carvalhais et al., 2014) or 96-well plates (for antimicrobial assays) (Oliveira et al., 2015). Of note, for confocal analysis, biofilms were formed on Nunc™ thermanox™ coverslips (Thermo Fisher Scientific) that were placed inside the wells. After 48 h of growth, biofilms were washed twice and either stained (for confocal analysis) or scraped from the plate bottom, in 0.9% NaCl, and submitted to a pulse of sonication of 10 s at 33% amplitude (Cole-Parmer), to dissociate cells clusters but without interfering with cell viability (Freitas et al., 2014). The amount of planktonic and biofilm cells was then quantified by OD_640nm_ and CFU counting. Moreover, for VBNC state-modulated biofilms, the total number of viable cells was also assessed by flow cytometry as previously optimized (Cerca et al., 2011b). The gating strategy is represented in **Supplementary Figure 1A**. Additionally, their structure was analyzed by confocal laser scanning microscopy (CLSM) using wheat germ agglutinin (WGA), which binds PNAG and allows the detection of the matrix, and DAPI, which stains nucleic acids and allows cells visualization (Cerca et al., 2006).

### Gene Expression

To quantify the expression levels of the genes cloned into the plasmid pRMC2, planktonic cultures induced for 4 h with 0.64 μg/ml ATC were used. For the quantification of gene expression in 48 hour-old biofilms, biofilm cells were collected as described above and, then, pooled together to decrease variability (Sousa et al., 2014). One milliliter of both planktonic or biofilm cells pool was used for RNA isolation with the kit ExtractMe RNA Bacteria & Yeast (Blirt S.A.) and as optimized before for *S. epidermidis* (França et al., 2012). RNA was treated with DNase and RNA concentration and purity were assessed using NanoDrop One (Thermo Fisher Scientific). Subsequently, RNA was reverse transcribed using the RevertAid H minus M-Mulv RT enzyme (Thermo Fisher Scientific) and quantitative PCR (qPCR) reactions prepared using Xpert Fast SYBR Mastermix (GRiSP). Finally, samples were run in a CFX96 thermal cycler (Bio-Rad) with the following cycle parameters: 95°C for 2 min, and 40 cycles of 95°C for 5 s, 60°C for 30 s. No-template and no-reverse transcriptase controls were included to evaluate, respectively, reagents and RNA contamination with genomic DNA. The absence of unspecific products and primer dimers was assessed by analysis of melting curves. Primers were designed with Primer3 (Untergasser et al., 2012) using *S. epidermidis* 1457 genome as the template (**Supplementary Table 2**).

### Antimicrobial Tolerance

The susceptibility to antibiotics was evaluated upon 6 and 24 h of incubation through CFU counting and by the reduction of tetrazolium salt XTT, which estimates cells metabolic activity, as described before (Cerca et al., 2005). Briefly, 2 ml of TSB supplemented with the peak serum concentrations (PSC) of vancomycin (40 μg/ml) (VWR), tetracycline (16 μg/ml), and rifampicin (10 μg/ml) (all purchased from Sigma-Aldrich) were inoculated with 1 × 10^7^ CFU/ml of bacteria in the stationary phase (24-hour growth). In the case of biofilms, 200 µl of TSB supplemented with PSC of the antibiotics under study were added to 48 hour-old biofilms grown in 96-well plates. Both planktonic and biofilm populations were incubated at 37°C and 120 rpm for up to 24 h.

### Survival in Human Blood and Plasma

Plasma was separated by centrifugation of whole blood for 20 min at 1,440*g* and 4°C. The co-incubation of bacteria with either whole blood, plasma or TSB supplemented with heparin (TSB + heparin, control) was performed as described before (França et al., 2016; Brás et al., 2020). Briefly, 50 µl of bacterial suspensions with 2 × 10^5^ CFU/ml were mixed with 450 µl of whole blood, plasma or TSB + heparin and incubated at 37°C and 80 rpm for up to 4 h. Cells culturability was assessed at the beginning of the assay (T = 0 h) and 1, 2, and 4 h after incubation.

### Dendritic Cells and Macrophages Differentiation

Peripheral blood mononuclear cells (PBMC) were isolated from buffy coats through density gradient centrifugation (1,200g for 15 min), using Histopaque 1077 (Sigma-Aldrich) in SepMate PBMC isolation tubes (Stemcell Technologies). CD14^+^ cells were isolated using anti-human CD14 MicroBeads (Miltenyi Biotec), according to manufacturer’s instructions and, then, suspended in complete RPMI medium—RPMI 1640 (Sigma-Aldrich) supplemented with 10% Fetal Bovine Serum (Biowest), 4 mM L-glutamine (Sigma-Aldrich), 10 mM HEPES (Sigma-Aldrich), and 50 μM *β*-mercaptoethanol (Merck) and seeded on 6-well culture plates at a concentration of 1 × 10^6^ cells/ml. Granulocyte–macrophage colony-stimulating factor (GM-CSF) (50 ng/ml) and macrophage colony-stimulating factor (M-CSF) (50 ng/ml) were used to differentiate monocytes into M1- and M2-like macrophages, respectively. To differentiate monocytes into dendritic cells (DC), GM-CSF (50 ng/ml) and interleukin-4 (50 ng/ml) were used. All cell cultures were incubated at 37°C with 5% CO_2_ for 7 days. Every 3 days, half of the cell culture medium was replaced with fresh complete RPMI with the respective growth factors.

### Infection of DC and M1/M2 Macrophages

On day 7, non-adherent and loosely adherent DC were harvested by gentle up and down pipetting movements. M1/M2-like macrophages were detached with 5 mM EDTA. Afterwards, cells were seeded in 96-well plates and infected with each strain using a multiplicity of infection (MOI) of 1:5 or 1:10 (human cell: bacteria) and incubated at 37°C and 5% CO_2_. After 2 h of incubation, 50 μg/ml gentamicin (AppliChem) was added to stop bacterial growth and the plates were again incubated at 37°C and 5% CO_2_ for 24 h. After the incubation period, the plates were centrifuged (300*g*, 10 min), the supernatants collected and stored at −80°C for cytokines quantification. In the case of DC, after supernatant collection, the cells were stained for flow cytometry cell surface activation detection.

### Quantification of Cytokine Production

Cytokine levels in cell culture supernatants were evaluated by sandwich ELISA using commercial kits, according to the manufacturer’s instructions (TNF-α, IL-6, IL-8, IL-10, and IL-12p70 DuoSet^®^ ELISA Development System—R&D Systems).

### Evaluation of DC Activation

To assess the expression of cell surface activation markers, DC were collected 24 h after infection with *S. epidermidis* strains, washed and incubated with fixable viability dye (eFluor 780) and with cell-surface antibodies, anti-human CD11c-APC conjugated (clone BU15), anti-human HLA-PECy7 conjugated (clone L243), anti-human CD83-FITC conjugated (clone HB15e), anti-human CD14-PE-conjugated (clone 61D3), anti-human CD80-BV510 conjugated (clone 2D10), and anti-human CD86-PECy5 conjugated (clone IT2.2), all purchased from eBiosciences. Cells were fixed with paraformaldehyde and resuspended in FACS buffer. Samples were analyzed by flow cytometry, using BD FACSCanto™ II and data analysis was performed using FlowJo™ 10.7.1 Software (BD Life Sciences), using the gating strategy represented in **Supplementary Figure 1B**.

### Ethics Statement

Human blood was collected in heparin-coated tubes (Vacuette), from adult healthy volunteers under a protocol approved by the Institutional Review Board of the University of Minho [SECVS 002/2014 (ADENDA)]. Buffy coats from healthy adult blood donors, used for M1/M2 macrophages and DC differentiation, were acquired at the Immunohemoterapy Department of Centro Hospitalar de São João (Porto, Portugal), under ethical approval of the service (Protocol reference 260/11). All procedures were performed in agreement with the Helsinki declaration and Oviedo convention and all donors gave written consent before blood collection.

### Statistical Analysis

Statistical analysis was performed using either unpaired T-test with Welch’s correction or One-way ANOVA with Tukey’s multiple comparisons test, using GraphPad Prism version 7 (Trial version, CA, USA). *P*-values below 0.05 were considered significant.

## Results

### *mazEF* Homologue Mutation and Complementation

The deletion of *mazEF* homologue in *S. epidermidis* strain 1457 was successfully achieved as confirmed by PCR and qPCR (**Supplementary Figure 2**), originating the mutant strain *ΔmazEF*. We then complemented the mutant with *mazEF* homologue sequence using the plasmid pRB473. The presence of the plasmid was confirmed by PCR (**Supplementary Figure 3A**) and the expression of the genes was confirmed by qPCR (**Supplementary Figure 2**). In addition, qPCR analysis confirmed that *mazEF* homologue deletion did not significantly interfere with *rsbU* regulation (Donegan and Cheung, 2009; Schuster et al., 2015) and that the plasmid pRB473 + *mazEF* restored *mazEF* homologue expression (**Supplementary Figure 2**). Moreover, the mutant strain was also complemented with *mazE*, *mazF* or *mazEF* homologous sequences using the inducible plasmid pRMC2 (**Supplementary Figure 3B**) required for the toxin–antitoxin experiments, further described below.

### Role of *mazEF* Homologue in VBNC State Induction in *S. epidermidis* 1457

**Figure 1:**
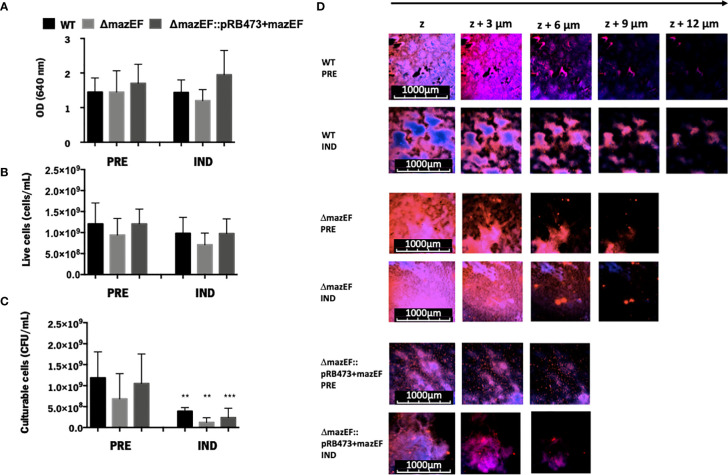
The role of *mazEF* homologue on the regulation of the VBNC state in *S. epidermidis* 1457 48-hour old biofilms. Quantification of *S. epidermidis* biofilms grown under VBNC inducing conditions in terms of **(A)** Total amount of cells (OD); **(B)** Concentration of live cells (flow cytometry); and **(C)** Concentration of culturable cells (CFU/ml). Results are represented as the mean + standard deviation of at least 3 independent experiments. Statistical analysis was performed with One-way ANOVA with Tukey’s multiple comparisons test, **p <0.01,***p <0.001. **(D)** Biofilm structure analysis assessed by CLSM. The images shown are representative of 2 independent experiments. PRE, Prevented VBNC; IND, Induced VBNC.

Based on the previous hypothesis that *mazEF* could be involved in VBNC state modulation in *S. epidermidis* biofilms, the proportions of VBNC cells in biofilms formed by the WT, *ΔmazEF*, and *ΔmazEF*::pRB473 strains were characterized using three techniques: OD, to evaluate the amount of total cells (**Figure 1A**), flow cytometry, to quantify total live cells (**Figure 1B**), and CFU counting, to determine the number of culturable cells (**Figure 1C**). No differences were observed in all strains tested, suggesting that *mazEF* did not have a significant role in VBNC state induction. We then assessed if there was any structural difference in the biofilms formed by these strains. However, as shown by the CLSM analysis (**Figure 1D**), no differences in biofilms structure were found.

### Role of *mazEF* Homologue in *S. epidermidis* 1457 Virulence Potential

Although our data do not support that *mazEF* homologue has an important role in VBNC state modulation in biofilms, we further explored the role of this operon in *S. epidermidis* 1457 general virulence. For that, the mutant strain complemented with the target genes and their natural promoter (*ΔmazEF*::pRB473+*mazEF*) was used to try to mimic the level of transcription in the WT strain.

**Figure 2:**
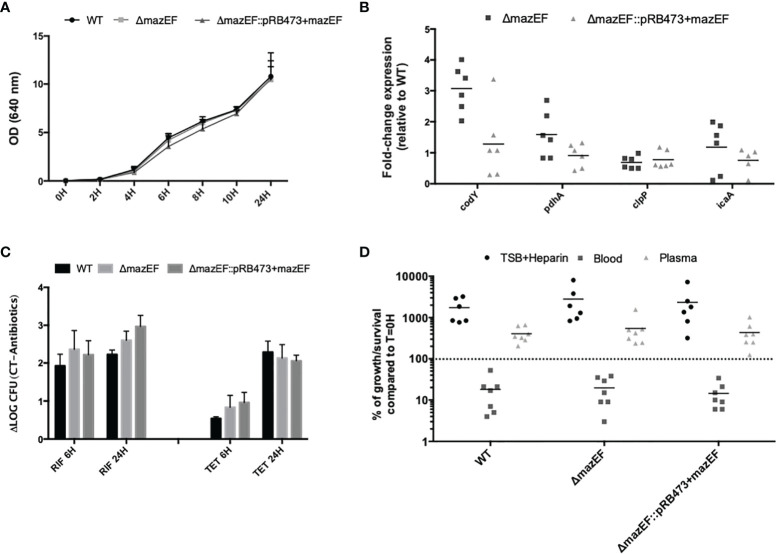
Characterization of the role of *mazEF* homologue constructs in *S. epidermidis* 1457 phenotype and virulence potential. **(A)** Growth curve of *S. epidermidis* strains as determined by optical density (OD 640 nm); **(B)** Fold-change expression in 48 hour-old biofilms formed by all strains. The values obtained in the WT strain were used as control. Each point represents a single experiment and the horizontal lines the mean of at least 5 independent experiments; **(C)** Effect of rifampicin (RIF) and tetracycline (TET) on the culturability of 48-hour old biofilm cells. Results are presented as the LOG difference between the control samples (no antibiotic) and the antibiotic-treated samples (ΔLOG); **(D)** Survival of *S. epidermidis* strains in human blood and plasma upon 4 h of incubation. Each point is relative to a different donor/assay and the horizontal lines represent the median of 6 independent experiments performed with different donors (3 females and 3 males). TSB supplemented with heparin (TSB + Heparin) was used to evaluate the effect of heparin on bacterial growth. Results **(A, C)** are presented as the mean + standard deviation of at least 3 independent assays. Statistical analysis was performed using One-way ANOVA with Tukey’s multiple comparisons test for all the assays.

As shown in **Figure 2A**, all strains presented a similar growth rate in the planktonic mode of growth. When analyzing the expression of genes putatively linked with VBNC cells formation (*pdhA*, *codY*, and *clpP*) (Carvalhais et al., 2014), and also related to biofilm formation (*icaA*) (Heilmann et al., 1996a; Heilmann et al., 1996b), we observed that the mutant had approximately a 3-fold increase in the expression of the gene *codY*, although some variability was observed among assays and no statistical significance was reached (**Figure 2B**). Regarding antimicrobial tolerance of biofilms (**Figure 2C**), minor differences in cells culturability were detected between the mutant and WT, but none were statistically significant. Interestingly, when evaluating biofilm cells metabolism, we observed that tetracycline was significantly more effective against the WT strain (**Supplementary Figure 4**). Furthermore, the same assays were repeated for planktonic cultures but, again, no substantial differences were found among strains (**Supplementary Figure 5**). Finally, when analyzing the ability to survive in human blood and plasma, similar results were obtained among strains at either earlier (1 and 2 h) (**Supplementary Figure 6**) or later (4 h) time points (**Figure 2D**).

**Figure 3:**
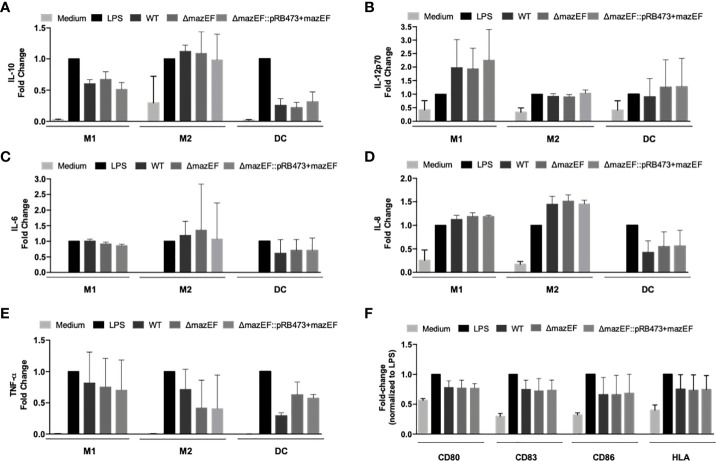
Effect of *mazEF* homologue deletion in the response of mononuclear phagocytes. **(A–E)** Quantification of the cytokines secreted by human monocyte-derived M1- and M2-type macrophages (M1 and M2) and dendritic cells (DC) in cell-culture supernatants upon incubation with *S. epidermidis* cells at a MOI of 1 M1/M2/DC:5 bacteria. **(F)** Cell surface expression of activation/maturation markers detected by flow cytometry on DC upon incubation with *S. epidermidis* cells at a MOI of 1 DC:5 bacteria. Cytokine levels and activation marker expression are presented as fold-changes to the respective values of positive control (LPS) samples. Bars correspond to means + standard deviation of at least 3 independent experiments, where each condition was set in duplicate. Statistical analysis was performed using One-way ANOVA with Tukey’s multiple comparison test. HLA, human leukocyte antigen.

Despite the lack of compelling differences in the ability of the strains to survive in human blood and plasma, we also assessed if the *mazEF* homologue could have an impact on the response of macrophages and DC when challenged with *S. epidermidis*. As shown in **Figure 3**, similar results were detected in the production of pro- (IL-12, IL-6, IL-8, and TNF) or anti-inflammatory (IL-10) cytokines by M1- and M2-like macrophages or DC when stimulated with different multiplicities of infection (MOI 1:5, **Figure 3** or MOI 1:10, **Supplementary Figure 7**).

### Role of *mazEF* Homologue as a Putative TA System

**Figure 4:**
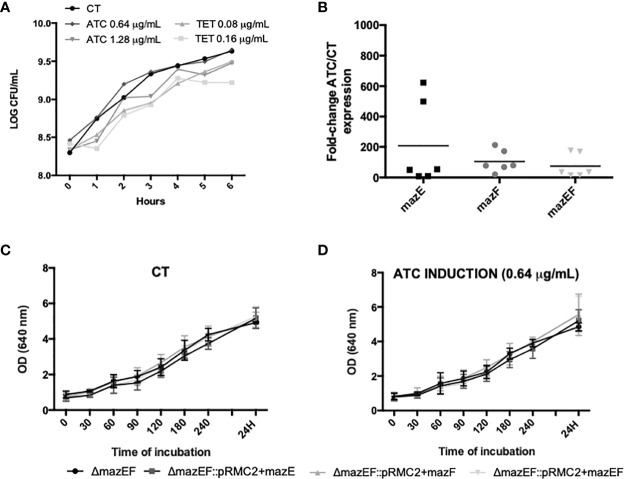
Study of the putative TA action of *mazEF* homologue. **(A)** Growth curve of *ΔmazEF* planktonic cells upon incubation with different concentrations of anhydrotetracycline (ATC) and tetracycline (TET). The control (CT) data refers to non-induced cells. The data presented are relative to a single assay; **(B)** Fold-change expression of the *mazEF* homologue genes upon 4 h of induction with ATC 0.64 μg/ml. *ΔmazEF* cells harboring the inducible plasmids but without induction were used as control. Each point represents a single experiment and the horizontal lines represent the mean of 6 independent experiments; **(C)** Optical density (OD) of the control strains; and **(D)** strains induced with 0.64 μg/ml of ATC. Results are represented as the mean + standard deviation of at least 3 independent assays **(C, D)**. Statistical analysis was completed with One-way ANOVA with Tukey’s multiple comparisons test **(B–D)**.

Since *mazEF* operon has been described, either experimentally or by homology, as a TA system in some staphylococcal species, we aimed to experimentally investigate if it has the same function in *S. epidermidis* 1457. After successful transformation of pRMC2 plasmids, the expression of the target genes was induced with either tetracycline (TET) or anhydrotetracycline (ATC), aiming to assess the best concentration to induce expression but without interfering with the culturability of *ΔmazEF* cells. While TET caused a slight reduction in the number of culturable cells compared to the control (no antibiotic), the ATC did not significantly affect cells culturability (**Figure 4A**). To ensure that *mazE/F/EF* homologues were being expressed under the selected inducing conditions (0.64 μg/ml of ATC), a qPCR analysis was performed and showed that *mazEF* homologous genes were being significantly expressed (**Figure 4B**). To analyze the putative role of *mazEF* as a TA system in *S. epidermidis*, the amount of total cells, as determined by OD, and the number of culturable cells, as determined by CFU counting, was assessed in the mutant and the complemented strains upon induction of *mazE/mazF/mazEF* homologues expression. However, in our conditions, no significant differences were observed in the OD (**Figures 4C, D**) or cells culturability (**Supplementary Table 3**).

## Discussion

The *mazEF* operon was initially identified as a TA system in *E. coli*, and then further confirmed in other gram-negative and gram-positive bacteria, with homology studies suggesting that this operon could have a TA function also in *S. epidermidis*. Importantly, although the homology between *S. epidermidis* 1457 *mazEF* homologue operon and *E. coli* is not very high (about 40% of nucleotide and 22 to 36% amino acid homology), greater homology was found with *S. aureus* strain Newman (**Supplementary Table 4**), where *mazEF* has been recently confirmed to function as a TA system (Ma et al., 2019). Interestingly, other biological roles have been linked with *mazEF*. Recently, *mazEF* increased expression was associated with *P. aeruginosa* and some staphylococcal species higher tolerance to gentamicin, ciprofloxacin, and clindamycin (Coskun et al., 2018). Furthermore, a role in biofilm formation and evasion from the host system was also described in *S. aureus* (Ma et al., 2019).

In *S. epidermidis*, preliminary evidence suggesting that *mazEF* homologue could play an important role in virulence came from previous transcriptomics studies, where it was suggested that the *mazEF* homologue could be involved in the regulation of the VBNC state in biofilms (Carvalhais et al., 2014). As such, we started by evaluating if the *mazEF* homologue mutant could impact the proportion of VBNC cells. VBNC cell formation in *S. epidermidis* biofilms is not universal, showing high variability among strains (Carvalhais et al., 2018). We recently confirmed that strain 1457 can form VBNC cells (Gaio et al., 2021), however, at a lower extent than previously tested strains (Carvalhais et al., 2018). Moreover, we observed that the expression of *mazEF* genes in biofilms grown under VBNC state inducing conditions was lower in 1457 than in other strains (Gaio et al., 2021). As such, it was not surprising that the lack of *mazEF* homologue did not significantly affect VBNC cells formation in *S. epidermidis* 1457, but we can’t exclude that in some clinical strains where the VBNC state is more pronounced, *mazEF* could play a role. Interestingly, the deletion of the *mazEF* homologue led to an increased expression of the gene *codY*, which has been also proposed to play a role in VBNC cells formation in *S. epidermidis* biofilms (Carvalhais et al., 2014). These results suggest that this gene may be compensating for the effect of the *mazEF* homologue absence, but this should be further explored. Other relevant genes, namely genes associated with biofilm formation, such as *icaA* and *rsbU* (Heilmann et al., 1996a; Heilmann et al., 1996b; Knobloch et al., 2004) were not affected by the *mazEF* homologue deletion.

We then further explored if the deletion of the *mazEF* homologue operon could influence other phenotypical traits in *S. epidermidis.* When assessing the response of planktonic and biofilm cells to vancomycin, rifampicin, and tetracycline, we observed that biofilm cells lacking *mazEF* homologue showed a significantly lower metabolic activity, after 6 h of treatment. This was an interesting result since it was previously shown that tetracycline might enhance the development of VBNC cells in *S. epidermidis* biofilms (Carvalhais et al., 2018). However, despite the change in metabolism, we did not observe any significant difference in bacterial culturability. Moreover, no differences in the bacterial survival in human blood or plasma were detected, as well as no relevant alterations in the levels of cytokines produced by M1- and M2-like macrophages or DC upon incubation with the distinct strains. This suggests that the deletion of *mazEF* homologue does not have a direct impact on the activation of the cells studied, which is usually driven by bacterial cell wall components.

Finally, considering the proximal relation and close homology to *S. aureus*, we also investigated the possible role of *mazEF* homologue operon as a TA module. In our experimental conditions, *mazEF* homologue did not seem to act as a TA system in *S. epidermidis* strain 1457. Despite the many similar traits between *S. aureus* and *S. epidermidis*, it was previously shown that the regulation of some important genes associated with biofilm formation is different, notwithstanding their significant homology (Cerca et al., 2008).

Altogether, in this brief report we performed the first characterization of a *mazEF* homologue mutant and demonstrated that, at least in strain 1457, the *mazEF* homologue does not seem to have a major role in *S. epidermidis* virulence potential.

## Data Availability Statement

The original contributions presented in the study are included in the article/**Supplementary Material**. Further inquiries can be directed to the corresponding author.

## Ethics Statement

Human blood was collected in heparin-coated tubes (Vacuette), from adult healthy volunteers under a protocol approved by the Institutional Review Board of the University of Minho (SECVS 002/2014 (ADENDA)). Buffy coats from healthy adult blood donors, used for M1/M2 macrophages and DC differentiation, were acquired at the Immunohemoterapy Department of Centro Hospitalar de São João (Porto, Portugal), under ethical approval of the service (Protocol reference 260/11). All procedures were performed in agreement with the Helsinki declaration and Oviedo convention and all donors gave written consent before blood collection.

## Author Contributions

Conceptualization, MV, NC, and AF. Investigation, VG and TL. Writing original draft, VG and AF. Writing—review and editing, NC, TL, and MV. Supervision, MV, NC, and AF. All authors contributed to the article and approved the submitted version.

## Funding

This work was supported by the Portuguese Foundation for Science and Technology (FCT) by the funder project PTDC/BIA-MOL/29553/2017, under the scope of COMPETE2020 (POCI-01-0145-FEDER-029553) and by the strategic funding unit UIDB/04469/2020. VG acknowledges the support of FCT individual fellowship [SFRH/BD/131452/2017].

## Conflict of Interest

The authors declare that the research was conducted in the absence of any commercial or financial relationships that could be construed as a potential conflict of interest.

## Publisher’s Note

All claims expressed in this article are solely those of the authors and do not necessarily represent those of their affiliated organizations, or those of the publisher, the editors and the reviewers. Any product that may be evaluated in this article, or claim that may be made by its manufacturer, is not guaranteed or endorsed by the publisher.
