# Supplementary material for: mazEF Homologue Has a Minor Role in Staphylococcus epidermidis 1457 Virulence Potential
Source: Front Cell Infect Microbiol. 2022 Jan 13;11:803134. doi: 10.3389/fcimb.2021.803134 (PMC8792614; doi:10.3389/fcimb.2021.803134)
Supplement: Supplementary file 1 [file DataSheet_1.docx]

Supplementary Material

*mazEF Homologue Has a Minor Role in Staphylococcus epidermidis 1457 Virulence Potential*

**Vânia Gaio^1^, Tânia Lima^2,3^, Manuel Vilanova^2,3,4^, Nuno Cerca^1^, Angela França^1*^**

^1^ Laboratory of Research in Biofilms Rosário Oliveira, Centre of Biological Engineering, University of Minho, Braga, Portugal

^2^ Instituto de Investigação e Inovação em Saúde, University of Porto, Porto, Portugal

^3^ IBMC - Instituto de Biologia Molecular e Celular, Universidade do Porto, Porto, Portugal

^4^ ICBAS - Instituto de Ciências Biomédicas de Abel Salazar, Universidade do Porto, Porto, Portugal

**Supplementary Table 1.** List of primers used for deletion and complementation processes. bp, base pairs; MCS, multiple cloning site.

| Designation | Forward sequence (5’-3’) * | Product size (bp) | Purpose |
| --- | --- | --- | --- |
| mazEF_P1 | GAATGCATGGAGTTGATGCTAA | 965 | Amplification of flanking regions (upstream) for *mazEF* homologue knockout construction |
| mazEF_P2 | GTTTTATAATAACAAATTTCCTAATCAGTG |  |  |
| mazEF_P3 | ttattataaaacACACTTATATAGTTGTCGATATGTCT | 1023 | Amplification of flanking regions (downstream) for *mazEF* homologue knockout construction |
| mazEF_P4 | TAACGTGTTTTGGGACTCACAC |  |  |
| mazEF_P1_Outside | TAGCACAACATTTAATGCGTCA | WT: 2719  KO: 2100  In combination with primer mazEF_P4 | *mazEF* homologue knockout confirmation |
| CPmazEF_F | GCTA**CCCGGG**ATGAAGAGGTTACTAACGAATTGTT | 869 | Amplification of *mazEF* sequence for complementation with pRB473 |
| CPmazEF_R | GCTA**CCCGGG**AAGCCTTAAAAACTCAAGACATCC |  |  |
| pIMAY_IM151 | TACATGTCAAGAATAAACTGCCAAAGC | 283 | Primers for screening of plasmid pIMAY MCS  (Monk et al., 2012) |
| pIMAY_IM152 | AATACCTGTGACGGAAGATCACTTCG |  |  |
| pRB473_Hind | CCAGTAATGACCTCAGAACTCC | 212 | Primers for screening of plamisd pRB473 MCS  (Brückner, 1992) |
| pRB473_Eco | CCCCAGGCGTTTAAGGGC |  |  |
| pRMC2_F | ATTCAGGCTGCGCAAC | 437 | Primers for screening of plasmid pRMC2 MCS (Corrigan & Foster, 2009) |
| pRMC2_R | TTGTTGACATTATATCATTG |  |  |
| CPmazEF_F_KpnI ¥ | GCTA**GGTACC**AAGGAGTGAAGTTATGGAGGTTCTTTTCATGTT | mazE: 223  mazF: 423  mazEF: 600 | Amplification of *mazEF* homologue genes for complementation with pRMC2 |
| CPmazE-R_BglII | GCTA**AGATCT**TAGATCATTCATTCTTTGAATTAGATATTAAAT |  |  |
| CPmazF_F_Kpn ¥ | GCTA**GGTACC**AAGGAGTGAAGTTATGGAATGATTAGAAGAGGA |  |  |
| CPmazEF_R_BglII | GCTA**AGATCT**TAGGTTTACTGCATATAATTATTTAAGATTT |  |  |

* Restriction enzymes recognition site (SmaI, KpnI and BglII) are underlined and in bold

**¥** Shine-Dalgarno sequence was added to these primers (underlined oligonucleotides)

**Supplementary Table 2** - List of primers used for qPCR analysis. bp, base pairs.

| Target gene | Forward sequence(5’-3’) | Reverse sequence  (3’-5’) | Product size  (bp) | Efficiency  (%) |
| --- | --- | --- | --- | --- |
| *16S rRNA* | GGGCTACACACGTGCTACAA | GTACAAGACCCGGGAACGTA | 176 | 100.0 |
| *gyrB* | GCATTTGGTACGGGTATTGG | CATCAACATCGGCATCAGTC | 88 | 93.0 |
| *mazEF* | GCAACAGAAGCTTTCCCGAT | CCCCTTGTTCAGACCCTTGA | 141 | 102.0 |
| *mazE* | CAAAATAGAAACCACAGTCTTGAAC | AGATATTAAATGTGATTCATTGCAATC | 138 | 91.8 |
| *mazF* | GAAGAGGAGATGTTTATTTAGCGG | CCCAAACTAATATCTAAGGCATTATC | 325 | 95.5 |

**Supplementary Table 3 –** Quantification of the number of culturable cells (LOG (CFU/mL)) of the mutant (*ΔmazEF*) and complemented strains (*ΔmazEF*::pRMC2+*mazE/mazF/mazEF)* and the difference of culturability (ΔLOG CT – ATC ) between the two conditions (CT: Uninduced strains; ATC: Strains induced with 0.64 μg/mL of ATC). CT, control; ATC, anhydrotetracycline.

| Condition | | *ΔmazEF* | *ΔmazEF*:: pRMC2+*mazE* | *ΔmazEF*:: pRMC2+*mazF* | *ΔmazEF*:: pRMC2+*mazEF* |
| --- | --- | --- | --- | --- | --- |
| 0H | **CT** | 8.03 ± 0.20 | 7.63 ± 0.13 | 8.06 ± 0.31 | 7.73 ± 0.09 |
|  | **ATC** | 8.08 ± 0.23 | 7.68 ± 0.07 | 7.99 ± 0.43 | 7.69 ± 0.08 |
|  | **ΔLOG CT-ATC** | **-0.05** | **-0.05** | **0.07** | **0.04** |
| 2H | **CT** | 8.54 ± 0.04 | 8.24 ± 0.14 | 8.50 ± 0.15 | 8.39 ± 0.17 |
|  | **ATC** | 8.47 ± 0.02 | 8.21 ± 0.09 | 8.40 ± 0.05 | 8.34 ± 0.24 |
|  | **ΔLOG CT-ATC** | **0.07** | **0.03** | **0.10** | **0.05** |
| 4H | **CT** | 8.77 ± 0.10 | 8.56 ± 0.09 | 8.74 ± 0.05 | 8.64 ± 0.04 |
|  | **ATC** | 8.74 ± 0.08 | 8.47 ± 0.04 | 8.68 ± 0.13 | 8.52 ± 0.05 |
|  | **ΔLOG CT-ATC** | **0.03** | **0.09** | **0.06** | **0.11** |
| 24H | **CT** | 9.20 ± 0.08 | 9.04 ± 0.31 | 9.24 ± 0.22 | 9.09 ± 0.29 |
|  | **ATC** | 9.26 ± 0.14 | 9.11 ± 0.36 | 9.29 ± 0.21 | 9.03 ± 0.29 |
|  | **ΔLOG CT-ATC** | **-0.06** | **-0.07** | **-0.05** | **0.06** |

**Supplementary Table 4** – Homology of *S. epidermidis mazE* and *mazF* homologous genes with sequences described as a TA system of the *mazEF* family in *S. aureus* and *E. coli.*

| Species/Strain | | *mazE* homology | *mazF* homology |
| --- | --- | --- | --- |
| *S. aureus Newman* | Nucleotide | 87.4% | 81.2% |
|  | Amino acid | 94.6% | 94.2% |
| *E. coli K12* | Nucleotide | 42.7% | 45.1% |
|  | Amino acid | 22.1% | 35.9% |

**
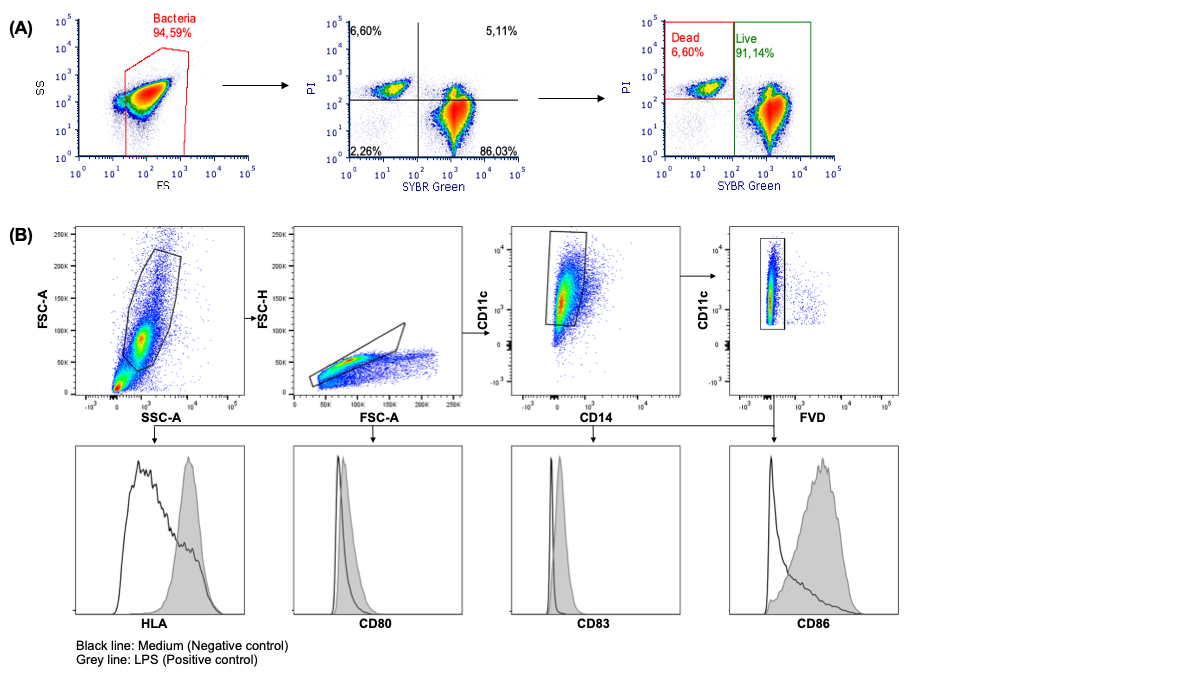
**

**Supplementary Figure 1.** **Representative gating strategy used on flow cytometry analysis. (A)** Representative gating used for the analysis of live cells within *S. epidermidis* populations grown under VBNC modulating conditions. Forward (FS) and side (SS) scatter signals were initially used to discriminate *S. epidermidis* cells (Bacteria gate) from background and debris. Then, the bacteria population was represented in a dot plot of SYBR green vs PI fluorescence and the number of live cells determined using both SYBR^+^/ PI^-^ and SYBR^+^/ PI^+^ populations, corresponding to live and potentially live cells, respectively. Dead cells (SYBR^-^/ PI^+^) and negligible debris (not stained, SYBR^-^/ PI^.^) were discarded. **(B)** Representative gating used for the analysis of activation makers expression upon incubation of dendritic cells (DC) with *S. epidermidis* strains. The samples were first filtered by size and complexity by representing them in a dot plot with forward (FSC-A) vs side (SSC-A) scatter. Then, using FSC-H (height) vs FSC-A (area) representation it was possible to select single cells and eliminate duplets for further analysis. Subsequently, the CD11^+^ population was selected at the same time that CD14^+^ cells (DC that did not differentiate) were excluded. Finally, dead cells that incorporated fixable viability dye (FVD) were also excluded from the analysis and the remaining CD11^+^ cells were used to measure the mean of intensity fluorescence (MIF) of the distinct surface markers.

**Supplementary Figure 2.** **Confirmation of *mazEF* homologue deletion and complementation with plasmid pRB473. (A)** Electrophoresis run upon PCR with genomic DNA of the selected clones. *S. epidermidis* 1457 WT expected size: 2700 bp; *S. epidermidis* 1457 *ΔmazEF* expected size: 2100 bp. **1.** ladder; **2-6.** Clones obtained after allelic replacement; **7.** Positive control (wild type (WT) gDNA); **8.** Negative control; Clone **#3** was selected for further studies. **(B)** Relative expression of *mazEF* homologue and *rsbU* genes of *S. epidermidis* 1457 constructs compared to the wild type (WT) strain. Gene expression was determined using 24h old planktonic cells and qPCR, as described in the material and methods section of the manucritpt. The results are displayed as the mean + standard deviation of 3 independent experiments. Statistical analysis was performed with Unpaired Welch’s T-test, ****p*<0.001.

**Supplementary Figure 3. Complementation of mutant strain with pRB473 and pRMC2 plasmids.** Result of electrophoresis run upon PCR with plasmids isolated from *S. epidermidis* 1457 *ΔmazEF*. **(A)** **1**. pRB473+*mazEF* (screening with primers CPmazEF_F and CpmazEF_R, expected size: 869 bp); **2.** 100 bp ladder; **3.** pRB473+*mazEF* (amplified with pRB473_Hind and pRB473_Eco screening primers, expected size: 1067 bp) **4.** Empty pRB473 plasmid (amplified with pRB473_Hind and pRB473_Eco screening primers, expected size: 212 bp); **(B)** pRMC2 plasmids, screening with primers pRMC2_F and pRMC2_R; **1.** pRMC2+*mazE* insert (expected size: 491 bp); **2.** 100bp ladder; **3.** pRMC2+*mazF* insert (expected size: 688 bp); **4.** 100bp ladder; **5.** pRMC2+*mazEF* insert (expected size: 868 bp);

**Supplementary Figure 4.** **Characterization of *mazEF* homologue genes constructs.** Effect of rifampicin (RIF) and tetracycline (TET) on the metabolic activity of 48 hours-old biofilm cells of *S. epidermidis* 1457 strains after 6 and 24 hours of incubation, assessed by the reduction of XTT (OD 490 nm). Results are presented as the difference between the control samples (CT, no antibiotic) and the antibiotic-treated samples (ΔOD). Data are presented as the mean + standard deviation of at least 3 independent experiments. Statistical analysis was performed with ANOVA with Tukey’s multiple comparisons test, **p*<0.05.

**Supplementary Figure 5.** **Characterization of *mazEF* homologues genes constructs.** Antimicrobial effect of rifampicin (RIF), tetracycline (TET) and vancomycin (VAN) on 24 hours-old planktonic cultures of *S. epidermidis* strains after 6 and 24 hours of incubation as determined by **(A)** culturability (LOG CFU/mL) and **(B)** metabolic activity (XTT). Results are presented as the mean + standard deviation of at least 3 independent experiments. Statistical analysis was performed using One-way ANOVA with Tukey’s multiple comparisons test.


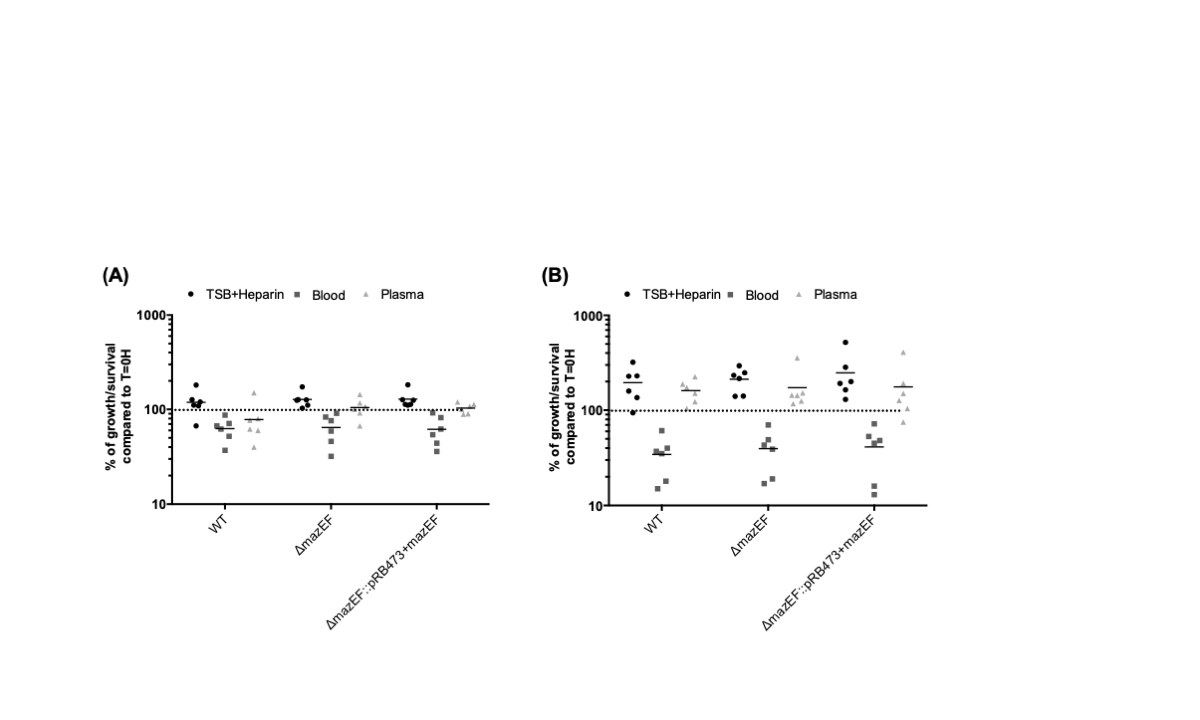


**Supplementary Figure 6.** **Characterization of *mazEF* homologue genes constructs.** Survival of *S. epidermidis* strains in human blood and plasma. TSB+Heparin was performed to evaluate the heparin effect on bacteria growth. Results are represented as the percentage of growth/survival (%) compared to T=0H upon incubation with TSB+Heparin, blood and plasma for **(A)** 1 hour and **(B)** 2 hours. The horizontal bars represent the mean of 6 independent experiments performed with blood collected from different donors (3 female and 3 male). Statistical analysis was performed with One-way ANOVA Multiple comparisons with Tukey’s test.

**
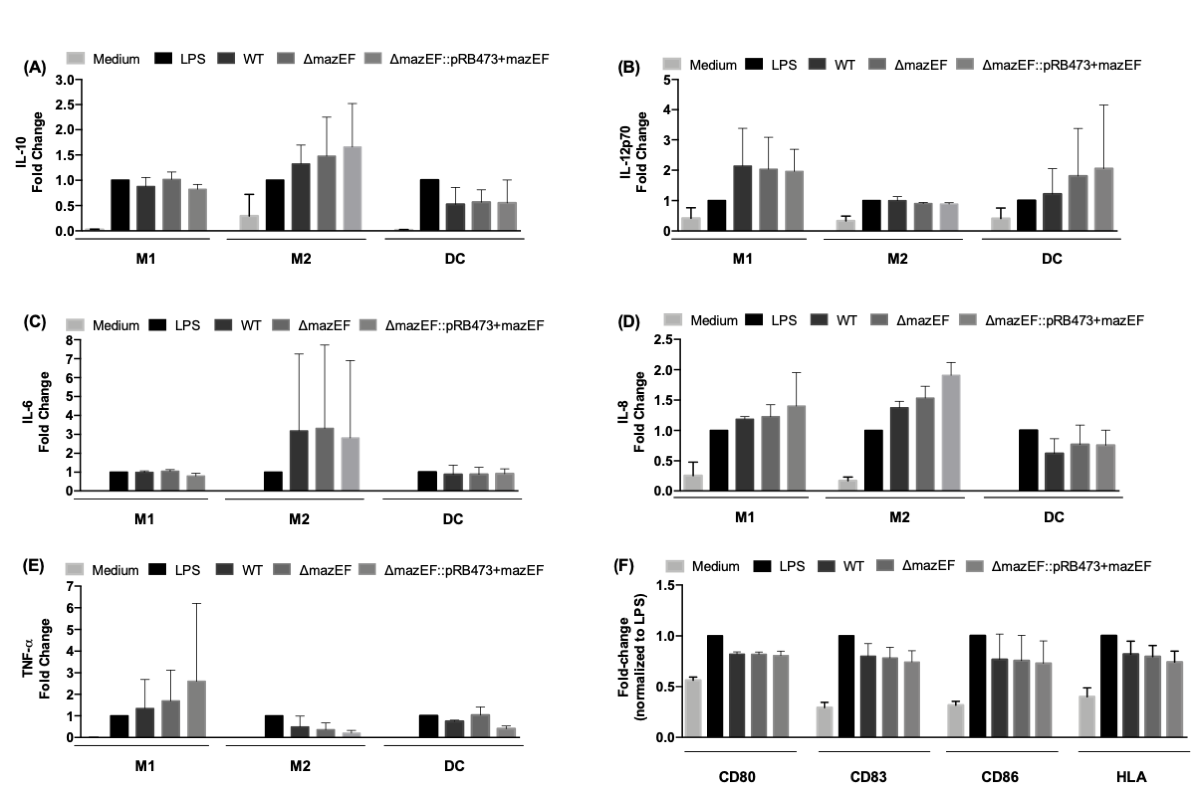
**

**Supplementary Figure 7 – Effect of *mazEF* homologue deletion in the response of mononuclear phagocytes**. **(A-E)** Quantification of the cytokines secreted by human monocyte-derived M1- and M2-type macrophages (M1 and M2) and dendritic cells (DC) in cell-culture supernatants upon incubation with *S. epidermidis* cells at a MOI of 1 M1/M2/DC to 10 bacteria. **(F)** Cell surface expression of activation/maturation markers detected by flow cytometry on DC upon incubation with *S. epidermidis* cells at a MOI of 1 DC : 10 bacteria. Cytokine levels and activation marker expression are presented as fold-changes to the respective values of positive control (LPS) samples. Bars correspond to the mean + standard deviation of at least 3 independent experiments, where each condition was performed in duplicate. Statistical analysis was performed using One-way ANOVA with Tukey’s multiple comparison test. HLA, human leukocyte antigen.
